# Topiramate (Topamax): Evolving Role in Weight Reduction Management: A Narrative Review

**DOI:** 10.3390/life13091845

**Published:** 2023-08-31

**Authors:** Irza Wajid, Alexis Vega, Katherine Thornhill, Jack Jenkins, Chandler Merriman, Debbie Chandler, Sahar Shekoohi, Elyse M. Cornett, Alan D. Kaye

**Affiliations:** 1 School of Medicine, Louisiana State University Health Sciences Center at New Orleans, New Orleans, LA 70112, USA; iwajid@lsuhsc.edu; 2 School of Medicine, Louisiana State University Health Sciences Center at Shreveport, Shreveport, LA 71103, USA; ajv001@lsuhs.edu (A.V.); kjt001@lsuhs.edu (K.T.); jsj001@lsuhs.edu (J.J.); cem002@lsuhs.edu (C.M.); 3 Department of Anesthesiology, Louisiana State University Health Sciences Center at Shreveport, Shreveport, LA 71103, USA; debbie.chandler@lsuhs.edu (D.C.); elyse.bradley@lsuhs.edu (E.M.C.); alan.kaye@lsuhs.edu (A.D.K.); 4 Department of Pharmacology, Toxicology, and Neurosciences, Louisiana State University Health Sciences Center at Shreveport, Shreveport, LA 71103, USA

**Keywords:** topiramate, weight loss, anti-epileptic, decreased neuronal excitation, migraine, essential tremors

## Abstract

Obesity has emerged as a widespread disease with epidemic proportions, necessitating effective management to enhance the overall health outcomes of patients. Medical intervention for weight loss becomes necessary when diet and exercise prove ineffective, and topiramate emerges as a potential treatment option for this global problem. Currently approved as an anti-epileptic and migraine prophylaxis medication, topiramate is frequently utilized as adjunctive therapy for patients with mood and eating disorders, as well as for alcohol use disorders. Its multifaceted mechanisms of action contribute to reducing neuronal excitation and enhancing neuronal inhibition. Given its variety of mechanisms, topiramate shows several off-label outcomes, including weight loss, for patients prescribed this medication. Although the specific mechanism of action concerning weight loss remains uncertain, various hypotheses have been reported. Notably, topiramate may contribute to weight loss by reducing calorie intake, decreasing fat gain, and lowering triglyceride and cholesterol levels. Additionally, its impact on reward pathways associated with food could play a role. Multiple clinical studies have supported the use of topiramate as a weight-loss medication. Notably, the medication demonstrates effectiveness in reducing body weight across different dosages and sustaining weight loss over time, outperforming alternative weight loss methods. Moreover, it was generally well-tolerated in clinical studies, with few side effects observed. In conclusion, topiramate offers promising potential as a weight loss solution and can be a valuable addition to the range of treatment options for combating obesity.

## 1. Introduction

Obesity is a global epidemic that continues to grow at an alarming rate. The World Health Organization (WHO) estimates that over one-third of the world’s population is overweight or obese. Obesity is associated with numerous health problems, including type 2 diabetes mellitus, respiratory problems, cardiovascular disease, psychological disorders, and cancer. The health risks associated with obesity are significant, and even a modest weight reduction can positively impact overall health and well-being [1].

To combat the obesity epidemic, various weight loss management techniques have been developed, ranging from lifestyle changes such as dietary modification and exercise to pharmaceutical interventions. While numerous medications have been developed to aid in weight loss, finding a safe and effective pharmacological intervention for obesity remains challenging.

One promising medication that has shown evidence of weight loss in patients is topiramate (TPM). TPM is currently approved as an anti-epileptic drug and has been shown to have weight loss effects in patients prescribed the medication. Several clinical studies have demonstrated that TPM can cause a significant reduction in overall body weight in patients [2].

Therefore, this investigation reviewed the clinical effects of TPM in treating obesity. This review aimed to summarize the current evidence supporting the use of TPM for weight loss and to identify any potential adverse effects associated with the medication. Overall, the findings suggest that TPM may be an effective pharmacological intervention for obesity. However, further research is needed to fully understand the medication’s effects and long-term safety.

## 2. Methods

This was a narrative review. The sources for this review are as follows: searching on PubMed, Google Scholar, Medline, and ScienceDirect using the following keywords: Topiramate, Weight loss, Anti-epileptic, Decreased Neuronal excitation, Migraine, Essential Tremors. Sources were accessed between March 2023 and July 2023.

## 3. Topiramate

TPM is an anti-epileptic drug indicated for seizure disorders and is commonly used as a mood stabilizer in psychiatric patients [3]. For almost two decades, between the mid-1970s, when carbamazepine and valproic acid were introduced, and 1993, when felbamate was approved, no new seizure drugs were developed for clinical use. More drugs have become available in recent years [4]. TPM was FDA-approved in 1996 as a monotherapy and adjunctive therapy for epilepsy and migraine treatment [3]. In 2012, TPM was approved for migraine prevention. Common off-label TPM uses include adjunctive therapy in bipolar disorder and other mood disorders, OCD, and PTSD. It has also been used in neuropathic pain, substance use disorders, eating disorders, and weight loss [3].

### 3.1. General Mechanism of Action

**Figure 1:**
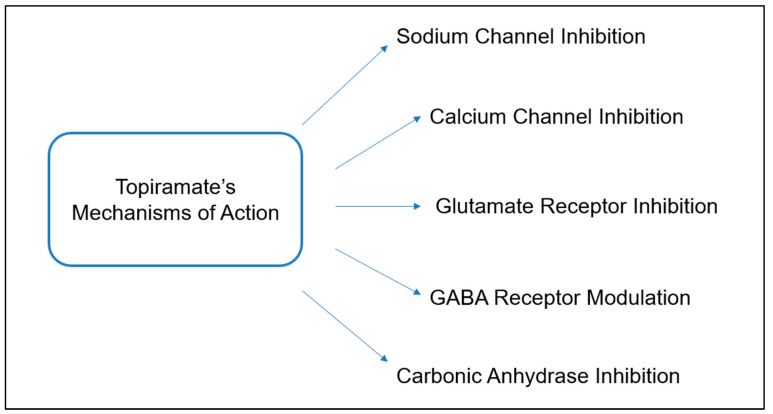
Topiramate mechanism of action. This figure was adapted with permission from [7].

Multiple physiological mechanisms are described for topiramate, which enhances its ability to treat a wide array of seizure types [4]. The main effects associated with topiramate are to reduce neuronal excitation and to enhance neuronal inhibition [4]. The drug possesses sodium-channel blocking activity, enhancement of cerebral GABA concentrations, and antagonism of the AMPA/kainite receptors, leading to decreased glutamate-mediated excitation and subsequent reduction in neuronal excitation [5]. TPM also increases the frequency of GABA-mediated chloride channel opening and increases potassium induction, further alleviating seizures by enhancing neuronal inhibition [4]. TPM is a less potent inhibitor than acetazolamide of carbonic anhydrase (Figure 1) [6].

### 3.2. Mechanism of Action in Relation to Weight Loss

Although the biological mechanism behind TPM-induced weight loss is not well-described, possible mechanisms include lowering calorie intake, decreased fat gain, and decreased triglycerides and cholesterol levels [8]. Studies show that TPM reduces energy deposition without changing food consumption by disrupting efficient energy utilization [9]. This suggests TPM can stimulate lipoprotein lipase in brown adipose tissue and skeletal muscle, increasing thermogenesis and substrate oxidation [10]. A review by Verotti et al. endorses the ability of TPM to induce weight loss, which is most significant in patients with a high BMI. Verotti et al. also reported the possibility that TPM reduces binge eating behavior by altering the reward pathway associated with the food [9].

Other sources have described that TPM may play a role in the gene encoding for neuropeptide Y, which influences the leptin pathway [9]. Leptin, a hormone involved in fat storage, is released by adipose tissue and binds receptors in the hypothalamus, which modulates the secretion of neuropeptide Y. Studies have described reduced leptin levels while patients were on topiramate, which directly correlated with weight reduction [11], A study in 2003 described reduced leptin levels in patients who were losing weight, and in patients who lost 10% or more body weight, leptin levels were reduced by 36% at three months [11]. Tremblay et al. report substantial body energy loss due to decreased macronutrient intake while patients were on topiramate, which led to lower body fat stores [12].

Overall, body weight and metabolism are also constantly regulated by signals from leptin and insulin in the hypothalamus. Pro-opiomelanocortin (POMC) neurons within the arcuate nucleus release melanocortin, which acts on the paraventricular hypothalamus to release thyrotropin-releasing hormone (TRH) and corticotropin-releasing hormone (CRH). Insulin and leptin act on this pathway to regulate metabolism [13,14]. Caricilli et al. concluded that fasted obese mice treated with TPM for 8 days showed increased mRNA levels of anorexigenic agents, such as POMC, TRH, and CRH, in the hypothalamus [14]. Increased activation of the pathway can also explain the overall weight reduction observed.

TPM has also been determined to lower fasting blood glucose, cholesterol, triglycerides, and HDL levels [11]. One study observed significant blood glucose reductions in overweight patients undergoing TPM therapy and more profound blood glucose reductions in obese patients [11]. TPM is a carbonic anhydrase inhibitor, which can influence the carbonic anhydrate enzymes involved in cellular lipogenesis [15]. Eliasson et al. noted that in obese patients diagnosed with Type 2 diabetes, treatment of topiramate for 11 months as an adjunctive showed significantly reduced body weight and improved glycemia control [16]. In 2005, Wilkes et al. studied female Zucker diabetic fatty rats treated with topiramate. Treatment decreased the glucose levels compared to untreated rats of the same weight [17]. The study determined that TPM treatment improved insulin sensitivity in diabetic mice and skeletal muscle [17]. These data were confirmed in a separate 2011 study showing that TPM induces weight loss and improves insulin sensitivity in dietary obese rats compared to sibutramine [18]. Khera et al.’s meta-analysis showed that patients treated with phentermine-topiramate, 15 mg/92 mg once daily, are associated with the highest probability of achieving at least a 5% weight loss [19]. Domecq et al.’s meta-analysis also showed that patients on topiramate for over 3 months experienced 3.7 kg of weight loss [20].

## 4. Topiramate Common Usage

One of the labeled indications for TPM is the treatment of epilepsy. A 2007 randomized but unblinded study investigated the effectiveness of lamotrigine, gabapentin, carbamazepine, oxcarbazepine, and TPM in treating partial epilepsy. The study found no significant difference in efficacy between these drugs for seizure control, though lamotrigine was better tolerated than the others. This study supports that TPM is an effective treatment for partial epilepsy [21]. A 2007 randomized but unblinded study by the same team investigated the effectiveness of valproate, lamotrigine, and the effectiveness of TPM in treating generalized and unclassifiable epilepsy. The study found that, while valproate was superior to topiramate, this was based not on efficacy but on tolerability. Valproate and TPM were equivalent, while lamotrigine was less effective. This study supports that topiramate is also an effective treatment for generalized and unclassifiable epilepsy [22].

A 2005 double-blind, randomized, placebo-controlled, parallel-group trial investigated the efficacy of topiramate as an adjunct therapy for patients with juvenile myoclonic epilepsy. The study found a 50% or more reduction of primarily generalized tonic-clonic seizures in 73% of patients compared to 18% in the placebo group. This study supports topiramate in treating juvenile myoclonic epilepsy [23]. A 1999 double-blind, randomized study investigated the efficacy of TPM in treating Lennox–Gastaut syndrome. The study found an improvement in seizure frequency of 52% in patients taking TPM compared to 38% in the placebo group. Additionally, there was a 15% reduction in drop attacks in the topiramate group compared to a 5% increase in the placebo group. Overall, this study supports TPM in treating seizures associated with Lennox–Gastaut syndrome [4].

The other labeled indication for TPM is migraine prophylaxis. A 2004 randomized, double-blinded, parallel-group trial investigated the difference in efficacy of TPM at 100 mg/day, 200 mg/day, and immediate-release propranolol 160 mg/day as an active control. The study found that TPM, when given at a dose of 100 mg/day, is effective in the metrics of a mean reduction in the average monthly migraine frequency, average monthly migraine days, rate of rescue medication, and responder rate compared to the placebo. This data shows that topiramate is an option for migraine prophylaxis [24]. A 2004 randomized, double-blind, placebo-controlled study investigated the efficacy of topiramate at 50 mg/day, 100 mg/day, or 200 mg/day doses. The study found that the monthly migraine frequency was decreased in the 100 mg/day and 200 mg/day groups compared to the placebo, with improvements occurring in the first treatment month. This study also concluded that TPM is indicated for migraine prophylaxis at 100 mg/day and 200 mg/day doses [25].

A 2015 meta-analysis evaluated information from studies about off-label TPM use for essential tremors. The study found that, compared to the placebo, TPM use led to significant improvements for patients measured by a change in the Fahn–Tolosa–Marin tremor rating scale and improvements in motor tasks/functions and function disability scores. Overall, this study demonstrates the efficacy of TPM’s off-label use in treating essential tremors [26].

A 2015 literature review compiled information from studies of off-label TPM use for alcohol use disorders. This review found that TPM significantly impacted reducing drinks per day, drinks per drinking day, and the number of heavy drinking days, as well as increasing the percentage of days abstinent. Overall, the review found that the current studies on the subject support that the off-label use of TPM is beneficial for treating alcohol use disorders. However, insufficient evidence supported its use in alcohol withdrawal syndrome [27].

The side effect profile of TPM is extensive and can be a barrier to patient use. One of the most distressing and unique side effects of TPM use is word-finding problems with slowed mental processing and attention and memory difficulties. Additionally, TPM is associated with fatigue, dizziness, somnolence, mood changes, and suicidal ideation. Related to inhibition of carbonic anhydrase, TPM has been known to cause paresthesia, renal calculi, metabolic acidosis, hypokalemia, and taste disturbance. Cognitive side effects are more frequent in patients using TPM for migraine prophylaxis than in epilepsy patients [28].

## 5. Clinical Studies

Bray et al. evaluated the efficacy and safety of TPM for weight reduction in obese and healthy participants [29]. It was a double-blind, randomized, placebo-controlled trial conducted with three hundred and eighty-five subjects aged 18–75 with a BMI of ≥30 to <50 kg/m^2^ or ≥27 to <50 kg/m^2^ if the subjects also had controlled hypertension and/or dyslipidemia. They were randomized into either a placebo group or treatment group of TPM at 60 mg, 96 mg, 192 mg, or 384 mg daily. The participant dosages were slowly increased by 16 mg weekly until they hit their target doses. After twenty-four weeks of treatment, all participants were tapered off by a 50% dose reduction. Importantly, all participants partook in similar lifestyle plans. The mean percent weight loss from baseline to week 24 was −2.6% in the placebo group and −5.0%, −4.8%, −6.3%, and −6.3% in the TPM groups. Common adverse events mainly consisted of paresthesia, somnolence, and concentration, which improved after dosage adjustments. In the placebo group, 11% of the participants withdrew due to adverse events vs. 21% in the TPM group. TPM provided a significantly greater weight reduction than the placebo across all treatment groups.

Moradi et al. evaluated the effect of TPM on weight loss in patients with type 2 diabetes. During 2008–2010, this 32-week randomized clinical trial of 69 participants was split into a placebo (30 patients) group and a TPM (39 patients) group [30]. Both groups were randomly assigned to participate in a nonpharmacological lifestyle intervention group. BMI, blood pressure, lipid profile, and glycosylated hemoglobin (HgA1c) were evaluated. Based on the 69 intended treated patients, the mean BMI changes were significantly higher (*p* = 0.006), the mean weight loss was significantly higher (*p* = 0.005), and the systolic blood pressure and glycosylated HgA1c were significantly decreased in the TPM group (*p* = 0.021 and *p* = 0.047). Topiramate was effective at stimulating weight loss and improving glycemic control in obese, diabetic patients.

Toplak et al. evaluated the safety and efficacy of TPM in obese patients with type 2 diabetes concurrently treated with metformin [31]. It was a multicenter, double-blind, placebo-controlled trial consisting of 646 obese men and women (aged 18–75 with a BMI of 27–50 kg/m^2^). Diet, exercise, and behavioral modification were adjusted, as well as maintaining the patients on a 600 kcal diet reduction based on their metabolic requirements. First, a 6-week single-blind run-in was used, followed by randomization to the placebo, 96 mg/day of TPM or 192 mg/day of TPM. After 8 weeks of titration, the participants used the treatment or placebo for 52 weeks. The mean percentage change in weight and HgA1c from baseline to week 24 was assessed. The placebo, TPM 96 mg group, and TPM 192 mg group lost 1.7%, 4.5%, and 6.5% of their baseline body weight. Both TPM groups had significant mean percentage weight changes with a *p* < 0.001. Additionally, the TPM groups appreciated significant decreases in systolic blood pressure. Topiramate proved effective for weight reduction and increased glycemic control in patients with metformin treatment for type 2 diabetes.

Ben-Menachem et al. inspected the predictors of weight loss in patients taking topiramate in combination with an anticonvulsant [32]. It was an uncontrolled, prospective trial in which topiramate was added to the patients’ medication regimens who were already taking anticonvulsants for partial-onset seizures. Thirty-eight patients were evaluated from baseline to three months and one year of topiramate treatment. The baseline weight was reduced by 82% at three months and 86% at one year. The mean body weight was reduced by 3.9% of the baseline at three months and 7.3% of the baseline at one year. In patients with a BMI > 30, the mean weight loss was 4.3% at three months and 10.9 kg at one year. For all patients, three months correlated to stronger reductions in caloric intake *p* = 0.02, and at one year, weight loss correlated stronger to those with a higher BMI (*p* = 0.0007). The results confirmed that topiramate caused significant weight loss and was sustainable for one year with treatment. Obese patients may experience greater weight loss with continued therapy longer than one year.

Astrup et al. examined the safety and efficacy of TPM, specifically in maintaining weight loss following a low-calorie diet [33]. The trial consisted of 701 obese subjects with a BMI between 30 to 50, aged 18–75 years. They received a low-calorie diet for eight weeks. Accordingly, those who lost greater than 8% of their initial weight received 96 mg/day and 192 mg/day or the placebo. The initial study was planned for sixty weeks but was cut short due to enhanced tolerability of the medication and a new controlled-release formulation for the patients’ ease. The efficacy was based on the 44 weeks of treatment or placebo. Around 80% of the enrolled patients lost over 8% of their initial body weight, and 293 were analyzed for efficacy. Subjects receiving TPM at 96 mg lost 15.4% of their initial weight by week 44, and subjects receiving 192 mg of TPM lost 16.5% by week 44. The placebo group lost 8.9% of their initial weight, which was significantly different with a *p* < 0.001. The placebo group regained weight after the run-in, unlike their TPM subjects, who continued to lose weight. Most adverse treatment events were related to minor central nervous system problems. After 44 weeks of treatment, TPM was well-tolerated overall and allowed the patients to maintain their initial weight loss and lose significantly more weight than the placebo group and/or low-calorie diet.

Tonstad et al. evaluated the safety and efficacy of TPM treatment in obese subjects with essential hypertension [34]. It was a randomized, placebo-controlled trial with 531 obese subjects (a BMI from 27 to 50) with an established HTN. After a 4-week, placebo, run-in period, the subjects were randomly given a placebo, 96 mg/day, or 192 mg/day of TPM. Originally, the 60-week study was cut short due to a new controlled-release formulation, and the efficacy was based on 28 weeks of treatment. The 96 mg and 192 mg groups had a weight loss of 5.9% and 6.5% from baseline, with a *p* < 0.001 for each compared to the placebo. Additionally, both treatment groups saw a decrease in the diastolic BP of 5.5 mmHg and 6.3 mmHg compared to 2.1 mmHg in the placebo (*p* < 0.015). Similar to previous studies, the adverse events included paresthesia, fatigue, and difficulty concentrating. TPM created clinically relevant body weight and blood pressure reduction effects while maintaining relatively mild adverse effects.

Wilding et al. investigated the long-term efficacy and safety of TPM in obese patients. It consisted of a randomized, double-blind, placebo-controlled study of 1289 subjects from 18 to 75 years old with a BMI between 27 to 50 with no comorbidities or a BMI between 27 to 50 with HTN and dyslipidemia [35]. Concurrent weight loss programs with nonpharmacological treatment were introduced to all patients. The efficacy was evaluated by patients who completed one year of their assigned dose of 96, 192, or 256 mg/day of TPM. The modified intention-to-treat group consisted of 854 subjects. At 60 weeks, the placebo group lost 1.7% of the baseline body weight compared to 7.0%, 9.1%, and 9.7% in the treatment groups (*p* < 0.001). Blood pressure, glucose, and insulin requirements were significantly reduced, with *p*-values less than 0.0001. TPM treatment for at least one year yielded clinically significant results for weight loss, blood pressure control, and glucose tolerance. See Table 1.

The combination of phentermine and TPM is used in conjunction with a low-calorie diet and regular exercise to aid in weight loss. It is also used in obese individuals who may also have diabetes, hypertension, excessive cholesterol, or cardiovascular disease. The FDA approved a weight loss pill containing phentermine and extended-release topiramate (Qsymia by Vivus) on 17 July 2012 [36,37]. Qsymia (formerly known as Qnexa) is indicated as an adjunct to a reduced-calorie diet and increased physical activity in patients with a body mass index (BMI) greater than 30 kg/m^2^ or a BMI of 27 kg/m^2^ or greater and at least one weight-related comorbidity (such as hypertension, dyslipidemia, diabetes, prediabetes, or abdominal obesity). Phentermine (α,α-dimethylphenethylamine HCl) is a centrally acting appetite suppressant. Although phentermine’s precise mechanism of weight loss is not explained in the FDA briefing, it may be inferred from the package insert that it functions as a sympathomimetic drug, which may suppress hunger and boost metabolism. Several clinical trials showed that doses of 3.75/23 mg (phentermine) and 15/92 mg (TPM) were more effective than the placebo over the course of 52 weeks, 56 weeks, and 108 weeks. Insomnia, irritability, anxiety, headache, attention disturbances, depression, parched mouth, and nephrolithiasis were the adverse events that led to the discontinuation of treatment in all three clinical trials. Initial phentermine/topiramate CR dosing for adults is 75/23 mg daily for 14 days [36,38]. The daily dosage should then be increased to 7.5/46 mg for 90 days. After 90 days, if a higher dose is required, it can be increased to 11.25/69 mg daily for 14 days, followed by 15/92 mg daily thereafter.

### 5.1. Absorption

TPM is well absorbed from the GIT, and maximal plasma concentrations are typically reached within two hours. The bioavailability exceeds 80 percent. Concomitant ingestion of food slows the time required to reach peak plasma concentrations but does not substantially alter the extent of absorption. Topiramate can, therefore, be administered regardless of mealtime. Topiramate exhibits linear and predictable pharmacokinetics over the recommended dosage range of 15 to 400 mg per day [3].

### 5.2. Distribution

TPM has 15% to 41% plasma protein–plasma protein binding. As the blood concentration of topiramate increases, the proportion of bound topiramate decreases.

### 5.3. Metabolism

The metabolism of TPM involves hydroxylation, hydrolysis, and glucuronidation.

### 5.4. Excretion

Topiramate is eliminated and unchanged as metabolites via renal excretion. Clearance of topiramate in adults ranges between 20 and 30 mL/min. The median half-life of plasma elimination is approximately 21 h. The elimination half-life varies with age and concurrent use of enzyme-inducing or enzyme-inhibiting medications. Patients taking concomitant enzyme-inducing pharmaceuticals, such as phenytoin, barbiturates, and carbamazepine, experience an increase in clearance [3].

### 5.5. Side Effects

The side effects associated with TPM for weight loss are paraesthesia, taste impairment, psychomotor disturbances, taste perversion, hypoaesthesia, difficulty concentrating, pruritus, insomnia, and dizziness [39].

## 6. Conclusions

Obesity is an increasing problem that is associated with several health diseases that are detrimental to the overall health of patients and is also associated with several health diseases that are detrimental to patients’ overall health. TPM is an anti-epileptic drug mainly used to treat epilepsy and migraine but can also aid in weight loss. At present, the clinical studies discussed in Table 1 show evidence that TPM can significantly aid in lowering overall body weight and should be considered a weight-loss medication. Further higher-quality research is necessary to determine the exact mechanism of action of TPM’s effects on weight loss and learn whether it can work as an additive or synergistic mechanism with other medications.

Several mechanisms of action of TPM in relation to weight loss have been proposed, which include changes in the reward pathway associated with food, stimulation of lipoprotein lipase, and decreased leptin and blood glucose levels. TPM can aid in lowering the baseline BMI and aid in improving glycemic control, especially in obese patients. As research continues to investigate TPM’s role in weight loss, current data are limited for the long-term therapy of TPM and its potential side effects on a patient’s overall health.

## Figures and Tables

**Table 1:** Summary of the clinical studies.

Author (Year)	Year	Groups Studied and Intervention	Results and Findings	Side Effects	Conclusions
Bray et al. [29]	2003	Randomized, double-blind, placebo-controlled, dose-ranging trial on the efficacy of TPM for weight loss in subjects aged 18–75 years with BMI ≥ 30 to <50 kg/m^2^ (n = 385). The subjects received either placebo, 64, 96, 192, or 384 mg of TPM daily.	All doses of TPM produced significant weight loss in comparison with placebo. Higher doses of TPM correspond to greater weight loss.	Side effects were most often CNS- and PNS-related. The most common side effects were paresthesia, memory difficulty, and fatigue. Side effect incidence was generally correlated to higher doses of TPM.	All studied TPM doses effectively reduced subjects’ body weights compared to placebo. Lower doses (64 mg and 96 mg) were better tolerated while still producing statistically significant reductions in the weight of the subjects.
Moradi et al. [30]	2013	Randomized, double-blind, placebo-controlled, parallel-group study on the efficacy of TPM for weight loss in subjects with documented DM2, a BMI between 27 and 50 kg/m^2^, and aged 18–75 years (n = 69). Subjects were treated with either a placebo or 150 mg of TPM per day.	The TPM treatment group achieved statistically significant decreases in weight compared to the placebo group. The treatment group’s systolic blood pressure and HbA_1c_ significantly decreased compared to the placebo group, and diastolic blood pressure showed no significant difference.	Twenty-four of the thirty-nine subjects in the treatment group reported side effects, the most common being paresthesia and memory problems.	TPM 150 mg/day can effectively reduce weight loss by around 5% in obese patients with DM2. Measurements of metabolic parameters related to obesity and DM2 also show improvement when treating subjects with TPM.
Toplak et al. [31]	2007	Randomized, double-blind, placebo-controlled multicenter trial on the efficacy of TPM for weight loss and HbA_1c_ reduction in subjects with DM2 on metformin monotherapy, a BMI ≥ 27 to <50 kg/m^2^, and aged 18–75 years (n = 646). Subjects were treated with a placebo, 96 mg/day of TPM, or 192 mg/day of TPM.	The 96 mg/day and 192 mg/day TPM treatment groups had statistically significant reductions in baseline bodyweight (4.5% and 6.5%, respectively) compared to placebo. TPM groups also had significant reductions in HbA_1c_ compared to the placebo.	Side effects were significantly more common in the treatment group. A total of 32% of subjects treated with TPM experienced paresthesia.	The addition of TPM to subjects with DM2 treated with metformin is effective in reducing body weight. Improved glycemic control was also observed in subjects taking TPM; side effects are common.
Ben-Menachem et al. [32]	2003	Uncontrolled, prospective trial of 38 in which topiramate was added to patient medication regimens who were also taking anticonvulsants for partial-onset seizure and monitored for 1 year.	Patients’ baseline weights and mean body weights were reduced. After 1 year for all patients, 3 months correlated to stronger reductions in caloric intake.	This study reported only fatigue and anxiousness—concentration/attention trouble, depression, paresthesia, language impairments, and dizziness. The study had no major adverse events or deaths. Seven patients discontinued topiramate due to treatment-emergent adverse effects. Nervousness, fatigue, and depression were the most common symptoms after topiramate discontinuation.	Topiramate caused significant weight loss and was sustainable for 1 year with treatment.
Astrup et al. [33]	2004	Randomized, double-blind, placebo-controlled, parallel-group study on the efficacy of TPM on maintaining weight loss after 8 weeks of a low-calorie diet. Obese subjects aged 18–75 years who lost ≥8% of their body weight received a placebo, 96 mg/day of TPM, or 192 mg/day of TPM (n = 701).	After weight loss of ≥8% across 8 weeks from diet, subjects treated with 96 mg/day or 192 mg/day of TPM had further weight decreases of 5.2 and 6.4%, respectively. These results were statistically significant compared to the weight gain of 1.8% experienced by the placebo group.	Paresthesia and fatigue were common side effects, but the effects were mainly mild to moderate.	TPM was effective for weight loss maintenance achieved previously by dieting in obese subjects. TPM also produced significant weight loss, while the placebo group experienced weight gain.
Tonstad et al. [34]	2005	Randomized, placebo-controlled study on TPM’s efficacy for weight and blood pressure reduction in obese subjects with documented hypertension (n = 531). Subjects were treated with a placebo, 96 mg/day of TPM, or 192 mg/day of TPM.	The 96 mg/day and 192 mg/day treatment groups had significant weight decreases compared to placebo. Diastolic blood pressure decreased in both treatment groups, but systolic blood pressure did not in comparison to the placebo.	Common adverse effects included paresthesia, fatigue, and concentration difficulty.	TPM 96 mg/day and 192 mg/day were effective for reducing weight in obese subjects compared to the placebo.
Wilding J. et al. [35]	2004	Randomized, double-blind, placebo-controlled, parallel-group study on the efficacy of TPM for weight loss in obese subjects without comorbidities. Subjects with hypertension and/or dyslipidemia were included. Subjects were treated with a placebo, 96 mg/day of TPM, 192 mg/day of TPM, or 256 mg/day of TPM (n = 1289) for 1 year.	Subjects in the groups treated with 96 mg/day, 192 mg/day, and 256 mg/day achieved a mean weight decrease of 7.0, 9.1, and 9.7%, respectively. These results were statistically significant compared to the placebo group.	The most common side effects are related to CNS, PNS, and psychiatric disorders. Paresthesia was reported by most subjects treated with TPM; however, it was generally well-tolerated.	TPM produced significant weight loss in obese subjects. Weight loss continued to week 60.

**Key:** Topiramate (TPM); body mass index (BMI); central nervous system (CNS); peripheral nervous system (PNS); hemoglobin A_1c_ (HbA_1c_); type II diabetes mellitus (DM2); controlled-release topiramate (CR-TPM).

## Data Availability

Data sharing is not applicable to this article as no datasets were generated or analyzed during the current study.
